# Therapeutic potential of matrix metalloproteinases in Duchenne muscular dystrophy

**DOI:** 10.3389/fcell.2014.00011

**Published:** 2014-04-01

**Authors:** Yuji Ogura, Marjan M. Tajrishi, Shuichi Sato, Sajedah M. Hindi, Ashok Kumar

**Affiliations:** Department of Anatomical Sciences and Neurobiology, University of Louisville School of MedicineLouisville, KY, USA

**Keywords:** skeletal muscle, matrix metalloproteinases, TIMPs, NF-κB, macrophages, inflammation, fibrosis

## Abstract

Matrix metalloproteinases (MMPs) are secreted proteinases that have physiologic roles in degradation and remodeling of extracellular matrix (ECM) in almost all tissues. However, their excessive production in disease conditions leads to many pathological features including tissue breakdown, inflammation, cell death, and fibrosis. Duchenne Muscular dystrophy (DMD) is a devastating genetic muscle disorder caused by partial or complete loss of cytoskeletal protein dystrophin. Progressive muscle wasting in DMD is accompanied by myofiber necrosis followed by cycles of regeneration and degeneration and inflammation that eventually result in replacement of myofiber by connective and adipose tissues. Emerging evidence suggests that gene expression and the activity of various MMPs are aberrantly regulated in muscle biopsies from DMD patients and in skeletal muscle of animal models of DMD. Moreover, a few studies employing genetic mouse models have revealed that different MMPs play distinct roles in disease progression in DMD. Modulation of the activity of MMPs improves myofiber regeneration and enhances the efficacy of transplantation and engraftment of muscle progenitor cells in dystrophic muscle in mouse models of DMD. Furthermore, recent reports also suggest that some MMPs especially MMP-9 can serve as a biomarker for diagnosis and prognosis of DMD. In this article, we provide a succinct overview of the regulation of various MMPs and their therapeutic importance in DMD.

## Introduction

**Figure 1:**
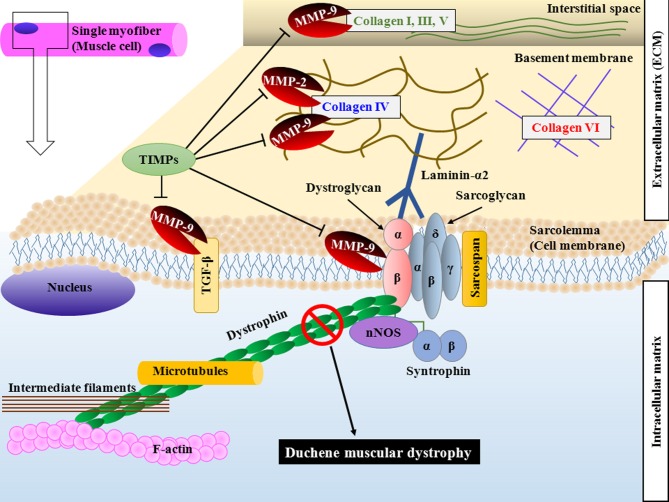
**Schematic representation of DGC and components of ECM targeted by MMPs in skeletal muscle.** Dystrophin stabilizes muscle cells by linking F-actin, intermediate filaments and microtubules to transmembrane DGC and ECM. Loss of dystrophin causes sarcolemma instability and increases susceptibility of sarcolemma to mechanical stress. MMPs are secreted by several cell types in skeletal muscle and cause proteolysis of specific substrates. MMP-2 and MMP-9 degrade collagen type IV. MMP-9 also degrades collagen type I, III, and V in interstitial space and cleaves β-dystroglycan, an important component of DGC. MMP-9 also converts membrane-bound latent TGF-β into active form by proteolytic cleavage. Binding of TIMPs inhibits the catalytic activity of MMPs. DGC, dystrophin-associated glycoprotein complex; ECM, extracellular matrix; MMP, matrix metalloproteinase; TIMP, tissue inhibitor of MMPs; TGF-β, transforming growth factor-β.

Skeletal muscle is composed of predominantly myofibers and responsible for almost all voluntary movements of the body. Myofibers are syncytium formed from the fusion of mononucleated myoblasts during embryonic development (Hindi et al., 2013a). Individual myofibers are surrounded by basement membrane, a thin layer of connective tissue composed of an internal basal lamina directly linked to sarcolemma and an external fibrillar lamina connected to extracellular matrix (ECM) in skeletal muscle (Sanes, 2003). Basement membrane and ECM are critical to maintaining structural integrity and normal function and to providing biochemical support to skeletal muscle (Sanes, 2003; Kjaer, 2004). Genetic studies in animals and humans have provided evidence that the loss of any of several components of ECM-basement membrane-sarcolemma-cytoskeleton network can lead to myopathy (Allamand and Campbell, 2000; Cohn and Campbell, 2000). Moreover, a number of extracellular or membrane-associated proteases, which get activated in specific disease states, can cause myopathy through proteolysis of the components of this network (Figure 1) (Nagase and Woessner, 1999; Kjaer, 2004; Zhong et al., 2006; Michaluk et al., 2007; Page-McCaw et al., 2007).

Muscular dystrophy refers to a group of genetic diseases which cause progressive degeneration of skeletal muscle fibers leading to severe pain, disability, and eventually death (Emery, 2002). The primary cause for various forms of muscular dystrophies is the mutations in individual genes that encode a wide variety of proteins including extracellular and transmembrane and membrane-associated proteins, cytoplasmic enzymes, and nuclear matrix proteins (Campbell, 1995; Blake et al., 2002). Duchenne muscular dystrophy (DMD) is the most common form of muscular dystrophy which afflicts 1 out of 3500 male births. DMD is caused by mutations in the DMD locus (Hoffman et al., 1987). Dystrophin is a critical component of a large complex known as dystrophin-associated glycoprotein complex (DGC) presents on plasma membrane of myofibers (Hoffman et al., 1987; Blake et al., 2002). Dystrophin stabilizes cells by linking actin filaments, intermediate filaments, and microtubules to transmembrane complexes (Prins et al., 2009). Loss of dystrophin leads to disruption of DGC complex, membrane instability, increased susceptibility to mechanical stress, and finally degeneration of myofibers (Rando, 2001). While skeletal muscle has extraordinary ability to regenerate in response to injury, the regenerative capacity of myofibers is compromised in DMD potentially due to exhaustion of satellite cells (muscle stem cells) as a result of chronic injury (Price et al., 2007).

It is now increasingly evident that the primary deficiency of dystrophin in skeletal and cardiac muscle results in the activation of several secondary processes such as calcium influx, infiltration of muscle tissue by inflammatory immune cells, accretion of proinflammatory and profibrogenic cytokines, activation of various proteolytic enzymes leading to ECM breakdown, and defects in clearance of damaged organelles through autophagy which all contribute to muscle wasting and disease progression in DMD (Shin et al., 2013). Accumulating evidence further suggests that DGC has an important signaling role in cardiac and skeletal muscle (Rando, 2001). Loss of dystrophin or other components of DGC leads to aberrant activation of several intracellular signaling pathways such as nuclear factor-kappa B (NF-κB), mitogen-activated protein kinases (MAPK), and phosphatidylinositol 3-kinase (PI3K)/Akt in skeletal muscle (Kumar and Boriek, 2003; Kumar et al., 2004; Acharyya et al., 2007; Bhatnagar and Kumar, 2010; Shi et al., 2013). Abnormal myogenic signaling has also been reported in other forms of muscular dystrophies that result from loss of nuclear membrane proteins or cytoplasmic enzymes suggesting that signaling defect is common to all types of muscular dystrophies which can potentially perpetuate dystrophic phenotype through modulating the activity of nuclear transcription factors and gene expression of specific effector proteins (Bhatnagar and Kumar, 2010).

Several genome-wide studies have suggested that the expression levels of a number of inflammatory molecules and regulators of ECM are highly disrupted in muscle biopsies from DMD patients and in skeletal muscle of animal models of DMD (Haslett et al., 2002; Porter et al., 2002; Kumar et al., 2010; Shin et al., 2013). Matrix metalloproteinases (MMPs) are one such class of molecules whose expression and activation is disrupted and appear to play a prominent role in disease progression in DMD (Table 1). In this article, we discuss the role of MMPs in pathogenesis of muscular dystrophy with emphasis on DMD.

**Table 1:** **Role of different MMPs in muscular dystrophy studied by pharmacological or genetic approaches**.

**Targeted MMP(s)**	**Approach to inhibit activity**	**Role in muscular dystrophy**	**References**
Pan-MMPs	Pharmacological	Cause myofiber injury	Girgenrath et al., 2009; Kumar et al., 2010; Percival et al., 2012; Pereira et al., 2012, 2014
		Mediate inflammation	
		Reduce levels of β-dystroglycan	
		Inhibit myofiber regeneration	
		Reduce levels of nNOS	
		Increase fibrosis	
		Reduce muscle force production	
		Activate MAPK and AP-1	
		Reduces life-span of dystrophic mice	
MMP-2	Genetic	Improves vascularization	Miyazaki et al., 2011
		Improves myofiber regeneration	
		Promotes fiber growth	
		Increases levels of VEGF-A	
MMP-9	Genetic	Causes myofiber injury	Li et al., 2009b; Dahiya et al., 2011a,b; Hindi et al., 2013b
		Increase inflammation	
		Reduces levels of β-dystroglycan and nNOS	
		Increases fibrosis	
		Reduces satellite cell number	
		Reduces myofiber regeneration	
		Reduces engraftment of progenitor cells	
		Increases levels of active TGF-β	
		Activates NF-κB and AP-1	
		Activates MAPK and Akt kinase	
		Inhibits notch signaling	
		Inhibits canonical Wnt signaling	
		Causes cardiomyopathy	
		Reduces skeletal and cardiac muscle function	
MMP-10	Genetic	Improves myofiber regeneration	Bobadilla et al., 2014
		Inhibits dystrophic phenotypes	

## Matrix metalloproteinases (MMPs)

MMPs are zinc-containing, calcium-dependent proteases that have an important role in ECM remodeling, inflammation, fibrosis, and activation of various latent cytokines, cell adhesion molecules, and growth factors in both physiological and pathological conditions (Vu and Werb, 2000; Page-McCaw et al., 2007). Based on their substrate specificity, MMPs are classified in four groups: the collagenases (MMP-1, -8, and -13), the gelatinases (MMP-2 and -9), the stromelysins (MMP-3, -10, and -11) and a heterogeneous group which contain matrilysin (MMP-7), metallo-elastase (MMP-12), enamelysin (MMP-20), endometase (MMP-26), and epilysin (MMP-28). MMPs are generally synthesized as secreted or transmembrane proenzymes which are processed to an active enzyme by the removal of an amino-terminal propeptide (Chakraborti et al., 2003). Once activated, MMPs are subject to inhibition by the tissue inhibitors of metalloproteinases (TIMPs) that bind to MMPs non-covalently (Vu and Werb, 2000; Page-McCaw et al., 2007). While the catalytic domain of MMPs is structurally similar, there are several differences in substrate specificity, cellular localization, membrane binding and regulation, making these a family of proteolytic enzymes having distinct functions (Nagase and Woessner, 1999; Vu and Werb, 2000; Page-McCaw et al., 2007).

MMP-2 (gelatinase A) and MMP-9 (gelatinase B) are the two major MMPs which degrade type IV collagen, an integral part of basement membrane (Turpeenniemi-Hujanen et al., 1985; Vu and Werb, 2000; Page-McCaw et al., 2007). MMP-9 is expressed during early embryonic development but is largely dormant in normal adult tissues. Gene expression and activation of MMP-9 are rapidly increased upon tissue injury suggesting its role in repair process, release of growth factors, and modulation of ECM for cell migration (Ram et al., 2006). By contrast, persistent presence of MMP-9 contributes to inflammation and tissue destruction in many disease states including chronic wounds (Ladwig et al., 2002; Rayment et al., 2008), heart failure (Chu et al., 2011), rheumatic arthritis (Munoz-Valle et al., 2003), fibrotic lung disease (Fukuda et al., 1998), dilated cardiomyopathy (Ducharme et al., 2000), multiple sclerosis (Fernandes et al., 2009), asthma (Corry et al., 2004), and cancer (Turpeenniemi-Hujanen et al., 1985; Chandler et al., 1997; Martin et al., 2008; Xu et al., 2010). MMP-2 which is constitutively expressed by many cell types including skeletal muscle has little regulation at the transcriptional level and most of the regulation takes place at the post-transcriptional level (Kherif et al., 1999; Nagase and Woessner, 1999; Chakraborti et al., 2003; Das et al., 2003). However, transcription of MMP-9 can be highly induced by numerous agents including cytokines, growth factors, cell–cell interaction, and cell-ECM adhesion molecules (Chakraborti et al., 2003). MMP-9 activity is regulated at various levels: gene transcription, synthesis, secretion, activation, inhibition, and glycosylation (Chakraborti et al., 2003). MMP-9 gene expression is under the control of a 2.2 kb upstream regulatory sequence containing binding sites for multiple transcription factors such as activator protein 1 (AP-1), NF-κB, specificity protein 1 (SP-1) which are conserved in different mammalian species (Gum et al., 1996; Nair and Boyd, 2005; Ram et al., 2006). Indeed, inflammatory cytokines TNF-α and TWEAK increase the gene expression of MMP-9 in skeletal muscle through activation of NF-κB and AP-1 transcription factors (Srivastava et al., 2007; Li et al., 2009a; Tajrishi et al., 2014).

Collagens are some of the most important proteolytic targets of MMPs in various tissues including skeletal muscle. Intramuscular connective tissue in skeletal muscle constitutes multiple collagen types (Kjaer, 2004). Whereas type IV collagen dominates the basement membrane adjacent to the sarcolemma, the fibrillar collagens type I and III (and to some extent type V) dominate the epi-, peri-, and endomysium in skeletal muscle (Koskinen et al., 2000; Ahtikoski et al., 2003). Even though collagen IV is the major substrate, MMP-9 can also degrade (but to a much lower extent) collagen I, III, and V present in skeletal muscle ECM (Vu and Werb, 2000; Mott and Werb, 2004). Furthermore, many other proteins such as laminin, fibronectin, entactin, elastin, and gelatin present in skeletal muscle ECM are other potential proteolytic targets for MMP-9 (Lewis et al., 2001; Chakraborti et al., 2003; Das et al., 2003). A few recent studies have demonstrated that MMP-9 cleaves β-dystroglycan, an important component of DGC in skeletal muscle (Zhong et al., 2006; Michaluk et al., 2007). In addition, it has been found that MMP-9 proteolytically converts latent membrane-bound transforming growth factor (TGF)-β into active protein which might be one of the mechanisms by which MMP-9 promotes fibrosis in diseased muscle (Figure 1) (Yu and Stamenkovic, 2000; Page-McCaw et al., 2007).

## Deregulation of MMPs in muscular dystrophy

There are several reports suggesting that gene expression and activation of various MMPs are deregulated in DMD patients. The first evidence about elevated activity of MMPs came from the studies by Sohar et al demonstrating that the serum levels of MMP-7 along with lysosomal cysteine proteinases (cathepsin H and L) are increased in DMD patients (Sohar et al., 1988). Since chronic degradation of ECM generally leads to fibrosis, von Moers et al studied expression of fibrolytic MMP-1 and MMP-2 and their physiological inhibitors TIMP-1 and TIMP-2 in muscle biopsies from DMD patients (von Moers et al., 2005). A significant increase in gene expression and activity of MMP-2 was evident in DMD muscle compared with controls. Furthermore, gene expression of TIMP-1 and TIMP-2 was increased in DMD muscle indicating deregulation of MMP activation (von Moers et al., 2005). Zanotti et al employed a cell culture model to investigate whether the expression of the components of ECM is altered in DMD muscle cells (Zanotti et al., 2007). This study showed that mRNA levels of several ECM molecules such as TGF-β1, myostatin, collagen I and VI, MMP-2, TIMP-1, and TIMP-2 were significantly higher whereas no significant differences were noticeable in mRNA levels of MMP-9 and TIMP-3 in DMD myotubes compared to normal myotubes (Zanotti et al., 2007). Interestingly, a progressive increase in levels of MMP-9 has recently been reported in serum of DMD patients (Nadarajah et al., 2011). Together, these findings suggest that while levels of MMP-9 along with other MMPs are increased, myofibers may not be the cellular source of MMP-9 in DMD patients (Zanotti et al., 2007; Nadarajah et al., 2011). Indeed, increased gene expression of various ECM components and MMPs has also been reported in cultured myofibroblasts from patients with DMD compared with healthy controls (Zanotti et al., 2010) further supporting the premise that multiple cell types may contribute to the increased levels of various MMPs in DMD patients.

Higher levels of MMPs have also been observed in animal models of muscular dystrophy. Fukushima et al studied MMPs and TIMPs in a canine X-linked muscular dystrophy in Japan (CXMDJ) model of DMD (Fukushima et al., 2007). They found that the expression levels and activation of MMP-2 and MMP-9 and their regulatory molecules such as MT1-MMP, TIMP-1, TIMP-2, and RECK (Reversion-inducing-cysteine-rich protein with kazal motifs) are significantly increased in dystrophic muscle of CXMDJ dogs compared to normal controls. MMP-2 and MMP-9 were predominantly localized in areas filled with degenerating and regenerating myofibers with cellular infiltrates further implying that inflammatory cells and fibroblasts are important sources of these gelatinases in dystrophic muscle (Fukushima et al., 2007).

Kherif et al provided initial evidence that gelatinolytic activity of MMP-2 and MMP-9 are increased in skeletal muscle of mdx (a mouse model of DMD) mice (Kherif et al., 1999). Since the activation of MMP-2 and MMP-9 is also increased in skeletal muscle after cardiotoxin-mediated injury, the authors suggested that these MMPs may have a role in myofiber regeneration (Kherif et al., 1999). However, it is notable that in normal skeletal muscle, the activities of MMP-2 and MMP-9 are increased only transiently after injury and return back to basal levels as the initial phases of regeneration subside. By contrast, MMP-2 and MMP-9 are constitutively expressed in dystrophic muscle of mdx mice which can lead to other changes in skeletal muscle microenvironment such as excessive remodeling of ECM and basement membrane leading to fibrosis. Roma et al. measured the levels of MMP-9, myosin heavy chain, utrophin, and β-dystroglycan protein in relation to the intensity of necrosis-regeneration at different age points in mdx mice (Roma et al., 2004). Protein levels of MMP-9 starts increasing at around 10 days which peaked between 25 and 60 days. During this period, the protein levels of utrophin and β-dystroglycan were significantly reduced in mdx mice indicating post-translational modification of structural protein in the myofibers of mdx mice (Roma et al., 2004). Interestingly, β-dystroglycan has now been found as an important target of MMP-9 leading to a characteristic 30 kDa fragment (Zhong et al., 2006; Michaluk et al., 2007). The 30 kDa fragment of β-dystroglycan has also been found to be increased in sarcoglycanopathy and DMD further suggesting that proteolysis of β-dystroglycan may contribute to skeletal muscle degeneration by disrupting the link between the ECM and sarcolemma (Matsumura et al., 2005). Increased production of MMP-9 with concomitant increase in the 30 kDa fragment of β-dystroglycan is also noticeable in diaphragm of mdx mice (Hnia et al., 2007). Moreover, immunohistochemical studies have suggested that similar to the CXMDJ model, MMP-9 is produced predominantly by macrophages and potentially other infiltrating cells in dystrophic muscle of mdx mice (Li et al., 2009b).

Recent studies have suggested that there is a cooperative interaction between various MMPs to induce tissue degeneration in multiple disease condition (Chakraborti et al., 2003). By performing real-time PCR array, we have reported that in addition to MMP-2 and MMP-9, the gene expression of several other MMPs such as MMP-3, -8, -10, -12, -13, -14, and -15 and TIMP-1 and -3 is increased in gastrocnemius muscle of mdx mice (Kumar et al., 2010). Our study also showed that pan-MMP activity was significantly elevated in dystrophic muscle of mdx compared with their normal counterparts (Kumar et al., 2010). Furthermore, protein levels and gelatinolytic activity of MMP-2 and MMP-9 are increased in cardiac muscle of mdx and mdx/utrophin double knockout mice (Dahiya et al., 2011a; Delfin et al., 2012).

## Targeting MMPs using pharmacological compounds

Since MMPs are also highly deregulated in cancer patients where they are linked to metastasis, several compounds have been developed to inhibit the activation of MMPs. Batimastat, a collagen peptide based hydroxamic acid, is among the first synthetic MMP inhibitors (MMPIs) developed to treat cancer (Fingleton, 2007, 2008). It mimics the site in the collagen substrate which is cleaved by MMPs. Batimastat functions by a competitive, reversible inhibition and effectively blocks the activities of MMP-1, MMP-2, MMP-3, MMP-7, MMP-8, MMP-9, and MMP-14 (Brown, 1995; Grams et al., 1995; Rasmussen and McCann, 1997). We studied the effects of batimastat in muscle pathology in mdx mice. Interestingly, chronic administration of batimastat in young mdx mice (starting at the age of 2-week) for a total of 5 weeks significantly reduced many pathological features in skeletal muscle such as infiltration of muscle tissue by inflammatory macrophages, gene expression of TNFα and cell adhesion molecules, myofiber necrosis, and fibrosis. The improvement in muscle pathology was also evident by the findings that diaphragm muscle force production in isometric contractions was significantly increased in batimastat-treated mdx mice compared to those treated with vehicle alone (Kumar et al., 2010). Consistent with proteolytic functions of MMPs, the levels of β-dystroglycan and neuronal nitric oxide synthase (nNOS) were improved in skeletal muscle of mdx mice on treatment with batimastat (Kumar et al., 2010). Furthermore, our study revealed that batimastat diminished the activation of MAPKs and transcription factor AP-1 in skeletal muscle of mdx mice. Although a short-term study, it provided the first evidence that pan-MMP inhibition is effective in reducing the severity of disease in a mouse model of DMD.

Tetracycline derivatives such as doxycycline and minocycline have been shown to non-specifically inhibit the activity of various proteases especially MMPs. Pereira et al. treated both young and aged mdx mice with doxycycline in drinking water for 1 month. This treatment significantly reduced myofiber necrosis, inflammation and fibrosis and improved muscle strength (Pereira et al., 2012). A more recent report from the same group documented that doxycycline reduces the levels of MMP-9 and TNF-α in dystrophic muscle of mdx mice (Pereira et al., 2014). Suramin, which inhibits binding of transforming growth factor-beta 1 (TGF-β1) to its receptor, has been also found to reduce myofiber necrosis and fibrosis and improve levels of β-dystroglycan potentially through reducing the levels of MMP-9 in dystrophic muscle of mdx mice (Taniguti et al., 2012).

Although activity of MMPs was not studied, tetracycline derivatives have also been shown to ameliorate myopathy in other models of muscular dystrophy. Girgenrath et al. have demonstrated that treatment with doxycycline or minocycline improves post-natal growth, delays the onset of hind-limb paralysis, and increases lifespan of the laminin-α2-null mice (a model of congenital muscular dystrophy) from ~32 to 70 days (Girgenrath et al., 2009). On similar lines, doxycycline improved skeletal muscle pathology in a mouse model of oculopharyngeal muscular dystrophy (Davies et al., 2005). Moreover, it has been recently reported that treatment of mdx mice with the phosphodiesterase 5 inhibitor sildenafil (Viagra®, Revatio®) reduces fibrosis and improves respiratory muscle contractility. Importantly, the levels of MMP-13 were significantly diminished in myofibers of mdx mice on treatment with sildenafil (Percival et al., 2012). Collectively, these studies suggest that pharmacological compounds which inhibit the activity of MMPs have therapeutic value in muscular dystrophy.

Even though broad spectrum MMP inhibitory compounds appear to improve myopathy in animal models of muscular dystrophy, it is noteworthy that so far no clinical trial using MMPIs has been considered as truly successful in cancer or other disease states because of deleterious side effects (Parsons et al., 1997; Nemunaitis et al., 1998; Macaulay et al., 1999; Bramhall et al., 2001, 2002a,b; Fingleton, 2007). However, it is also notable that the first generation MMPIs including batimastat and its derivatives were small peptide mimics that chelate the zinc ion and block the function of the enzymes. Treatment with these MMPIs led to the development of a musculoskeletal syndrome (MSS) that reduced the overall quality of life and withdrawal of patients from clinical trials (Brown, 1999; Dove, 2002; Fingleton, 2007, 2008). While it is unambiguous that MMPs represent one of the most important therapeutic targets for various tissue degenerative disorders (Brown, 1999; Dove, 2002; Fingleton, 2007, 2008), multiple reasons have been cited for the failure of the first generation broad spectrum MMPIs in clinical trials. A current theory is that the majority of the side effects associated with MMPIs in clinical trials are predominantly related to off-target metal (Zn and Fe) chelation by the first generation MMPIs (Renkiewicz et al., 2003). Strong evidence that MSS side effects are not related to MMP inhibition *per se* comes from the use of other drugs which do not cause metal chelation. A number of compounds have been reported to have the ability to block the activity or expression of MMPs including bisphosphonates (Giraudo et al., 2004; Ferretti et al., 2005), statins (Wilson et al., 2005; Yasuda et al., 2007), and antibiotics (Acharya et al., 2004). So far there is no evidence that treatment with any of these drugs is associated with MSS suggesting that better designed MMPIs would be more effective in clinical trials (Acharya et al., 2004). Because of the high levels of similarity in the catalytic domain of MMPs, it has been a great challenge to develop drugs which can inhibit the activity of a specific MMP. However, there can be other potential approaches to inhibit MMPs once their specific activators and regulators are identified. For example, it has been recently shown that osteopontin up-regulates the expression of MMP-9 in skeletal muscle of mice. Treatment with osteopontin neutralizing antibody reduced the levels of MMP-9 in dystrophic muscle of mdx mice (Dahiya et al., 2011a). Small molecules such as microRNAs represent another class of molecules which can modulate the levels of specific MMPs in disease conditions (Xu et al., 2012; Asuthkar et al., 2013; Wu et al., 2013).

## Targeting MMPs using genetic approaches

MMP-9 is highly overexpressed in dystrophic muscle of mdx mice. By crossing with *Mmp9*-knockout mice, we investigated the role of MMP-9 in skeletal and cardiac muscle pathology in mdx mice. Our analysis showed that heterozygous or homozygous deletion of *Mmp9* gene dramatically reduced serum levels of creatine kinase (a marker of muscle injury), inflammation, fibrosis and improved skeletal muscle structure and function and myofiber regeneration in 8-week old mdx mice (Li et al., 2009b). Furthermore, genetic ablation of MMP-9 also diminished serum levels of creatine kinase and improved muscle structure in 1-year old mdx mice indicating that the continued inhibition of MMP-9 is effective in reducing muscle injury in mdx mice (Dahiya et al., 2011b). Although the exact mechanisms by which excessive production of MMP-9 causes myopathy in mdx mice remain unclear, our analysis showed that the inhibition of MMP-9 increased the protein levels of β-dystroglycan and nNOS and reduced the amounts of active form of TGF-β in myofibers of mdx mice. Therefore, it is reasonable to speculate that the inhibition of MMP-9 improves the integrity of DGC on sarcolemma by preventing excessive degradation of its components. Since TGF-β increases fibrosis in skeletal muscle, diminished levels of active TGF-β1 upon inhibition of MMP-9 may be a potential mechanism for amelioration of fibrosis in dystrophic muscle of mdx mice (Li et al., 2009b).

**Figure 2:**
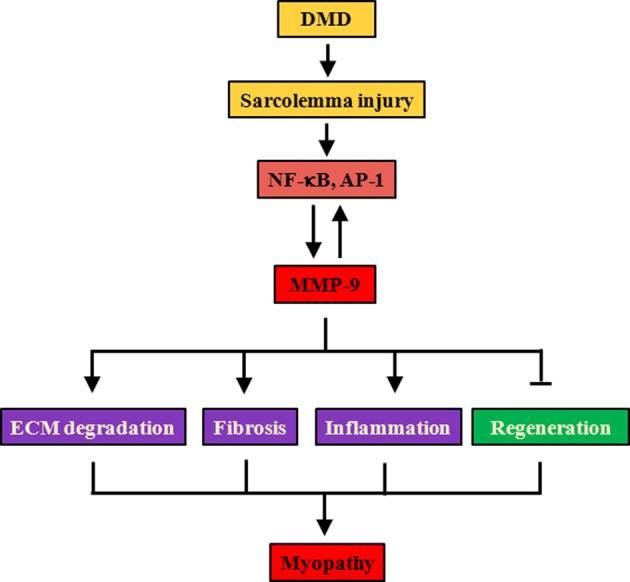
**Proposed mechanisms of action of MMP-9 in DMD.** Lack of dystrophin leads to destabilization of DGC, increased susceptibility to contraction-induced injury, and finally degeneration/regeneration of myofibers. Cycles of fiber degeneration and regeneration augment inflammatory response with concomitant activation of proinflammatory transcription factors NF-κB and AP-1. NF-κB, and AP-1 augments the gene expression of MMP-9. Increased production of MMP-9 also induces NF-κB and AP-1 activity potentially through proteolytic activation of various latent cytokines. Persistent presence of high levels of MMP-9 causes fiber necrosis, inflammation, and fibrosis and interferes with regeneration of damaged myofibers leading to myopathy. AP-1, activator protein 1; DGC, dystrophin-associated glycoprotein complex; DMD, Duchenne muscular dystrophy; MMP-9, matrix metalloproteinase-9; NF-κB, nuclear factor-kappa B.

Intriguingly, deletion of a single allele of *Mmp9* gene was sufficient to drastically reduce the frequency of macrophages (also a major source of MMP-9) and protein levels of MMP-9 by ~80% in dystrophic muscle of mdx mice. These findings suggest that MMP-9 induces its own expression through a positive feed-back mechanism (Figure 2). Indeed, we have reported that the inhibition of MMP-9 diminishes the activation of NF-κB and AP-1 in dystrophic muscle of mdx mice (Li et al., 2009b) which is in agreement with published reports that the gene expression of MMP-9 is regulated by NF-κB and AP-1 (Chakraborti et al., 2003; Srivastava et al., 2007; Li et al., 2009a). A number of studies have now shown that the activity of NF-κB is increased in muscle biopsies and in skeletal muscle of animal models of DMD (Acharyya et al., 2007; Bhatnagar and Kumar, 2010). Furthermore, inhibition of NF-κB using either genetic or pharmacological approaches ameliorates skeletal muscle pathology in models of DMD (Acharyya et al., 2007; Li et al., 2008; Bhatnagar and Kumar, 2010). MMP-9 appears to be one of the downstream effector molecules of NF-κB evident by the findings that treatment with peptide inhibitor of NF-κB drastically reduced the levels of MMP-9 in diaphragm of mdx mice (Li et al., 2009b). A cooperative interaction between NF-κB and MMP-9 in pathogenesis of mdx mice is also supported by the findings that activation of NF-κB and increases in the gene expression of MMP-9 follow a similar pattern in skeletal muscle of mdx mice (Kumar and Boriek, 2003; Acharyya et al., 2007; Li et al., 2009b). This notion is further supported by the findings that L-arginine, which improves myopathy in mdx mice, diminishes the expression of inflammatory cytokines and levels of NF-κB and MMP-9 in dystrophic muscle (Hnia et al., 2008).

There is a plethora of literature demonstrating that the abundance of many MMPs including MMP-9 is increased in response to a variety of cardiac insults resulting in cardiomyopathy (Tyagi et al., 1996; Halade et al., 2013). Targeted deletion of MMP-9 in mice has been found to attenuate coronary artery ligation-induced left ventricle enlargement and accumulation of collagens, suggesting a role for MMP-9 in cardiac remodeling after ischemic injury (Ducharme et al., 2000). Studies from our group and others have shown that the levels of MMP-9 are also increased in cardiac muscle of mdx and mdx/utrophin double knockout mice (Dahiya et al., 2011a; Delfin et al., 2012). We examined the role of MMP-9 in the development of cardiomyopathy in mdx mice. Ablation of MMP-9 attenuated injury, inflammation, left ventricle dilation, and fibrosis leading to improvement in heart function in 1-year old mdx mice. Ablation of MMP-9 also diminished the activation of extracellular-regulated kinase 1/2 and Akt/protein kinase B (PKB) in the cardiac muscle of mdx mice. Furthermore, inhibition of MMP-9 suppressed the gene expression of MMP-3 and MMP-12 in the heart of mdx mice which is further suggestive of a cooperative interaction between various MMPs in the settings of muscular dystrophy.

Although the mechanisms leading to the increased levels of MMP-9 remain unknown, proinflammatory cytokines whose abundance is increased in mdx mice, appear to be some of the potential stimuli for up-regulation in MMP-9 levels in skeletal and cardiac muscle in muscular dystrophy (Acharyya et al., 2007; Li et al., 2009a; Shin et al., 2013). As mentioned above, a recent study has demonstrated that protein levels of osteopontin (an important regulator of immune response) are significantly increased in circulation and dystrophic muscle of mdx mice and genetic ablation of osteopontin attenuates fibrosis in mdx mice (Vetrone et al., 2009). Neutralizing antibody against osteopontin reduced the amounts of MMP-9 in dystrophic muscle of mdx mice suggesting that osteopontin contributes, at least partly, to the increased amounts of MMP-9 in cardiac and skeletal muscle of mdx mice (Dahiya et al., 2011a).

In contrast to the role of MMP-9 in mdx mice, it has been reported that the forced expression of MMP-9 increases vascularization and reduces collagen deposition in skeletal muscle of 12-month old α-sarcoglycan-deficient mice, a mouse model of limb griddle muscular dystrophy (Gargioli et al., 2008). However, in this study, placenta growth factor was also used in addition to MMP-9 (Gargioli et al., 2008). Moreover, it is likely that a specific MMP may have different roles in various types of muscular dystrophy and it is the muscle microenvironment which dictates whether the inhibition of a MMP will provide beneficial or deleterious effects.

Levels of MMP-2 are also increased in dystrophic muscle of mdx mice (Kherif et al., 1999; Li et al., 2009b). Miyazaki et al have reported that genetic ablation of MMP-2 in mdx mice inhibits fiber growth and angiogenesis in skeletal muscle potentially through diminishing the levels of vascular endothelial growth factor-A, an important angiogenesis-related factor. Since the levels of β-dystroglycan remained unaffected, it is likely that only MMP-9 but not MMP-2 is involved in proteolysis of β-dystroglycan in dystrophic muscle (Miyazaki et al., 2011). The role of MMP-10 in pathogenesis of mdx mice has also now been investigated using genetic approaches (Bobadilla et al., 2014). Similar to MMP-2, MMP-10 also has a positive impact on skeletal muscle regeneration in mdx mice. The dystrophic phenotypes are exaggerated by the deletion of *Mmp10* gene in mdx mice. Moreover, knockdown of MMP-10 reduced myofiber regeneration whereas treatment with recombinant MMP-10 protein augmented myofiber repair in mdx mice (Bobadilla et al., 2014). Altogether, these studies using knockout mouse models have highlighted that not all MMPs are involved in pathogenesis of muscular dystrophy and some of them may be activated as a part of compensatory mechanism to counter muscle damage. These findings further emphasize the importance of understanding the specific roles of various MMPs and necessity to develop specific inhibitors for different MMPs before considering them as therapeutic targets for muscular dystrophy.

## MMPs and stem-cell based therapies for DMD

Recent advancements in stem cell-based therapies have suggested that the transplantation of stem cells including satellite cells can be an important approach for the introduction of functional dystrophin protein in DMD patients (Dezawa et al., 2005; Sampaolesi et al., 2006; Cerletti et al., 2008; Darabi et al., 2008). However, one of the drawbacks of this approach is that the transplantation of satellite cells into muscle tissue typically results in poor engraftment and death of significant amounts of cells, resulting in little repair of the diseased muscle (Darabi et al., 2008). The limitation in migration of transplanted cells in skeletal muscle is also another issue which could be attributed to the presence of excessive fibrosis and ECM abnormalities (Darabi et al., 2008). Approaches which reduce secondary changes such as inflammatory response and fibrosis prior to transplantation can potentially improve the functional engraftment of transplanted cells in dystrophic muscles (Qu et al., 1998). In fact, a few studies have shown that the attenuation of inflammation in dystrophic muscle improves the effectiveness of cellular therapy in models of DMD (Qu et al., 1998; Darabi et al., 2008).

Since MMPs play a prominent role in cell migration and invasion, Pichavant et al investigated the effects of overexpression of MMP-9 in muscle precursor cells (MPCs) and their transplantation in skeletal muscle of mice (Pichavant et al., 2011). They found that overexpression of MMP-9 did not affect the proliferation, migration, or differentiation; however, it enhanced the invasion capacity of MPCs *in vitro*. When MMP-9 overexpressing MPCs were transplanted in Reg1-null mice, an improvement in MPC transplantation and migration was noticeable suggesting that MMP-9 causes ECM remodeling leading to enhanced transplantation in healthy muscle. We have also reported that skeletal muscle-specific transgenic overexpression of a constitutively active mutant of MMP-9 causes hypertrophy and improves myofiber regeneration upon cardiotoxin-mediated injury (Dahiya et al., 2011b). However, it is notable that in these studies, disease-free mice were used and MMP-9 was overexpressed by either transplanted MPCs or myofibers which may not be sufficient to cause the same extent of ECM degradation and fibrosis that are observed in dystrophic muscle from mdx mice (Pichavant et al., 2011).

We investigated the role of MMP-9 in engraftment of MPCs in dystrophic muscle of mdx mice (Hindi et al., 2013b). Our study showed that the suppression of MMP-9 levels in dystrophic muscle of recipient mdx mice using genetic approach dramatically improves the engraftment of transplanted wild-type MPCs resulting in the expression of dystrophin protein in myofibers of mdx mice (Hindi et al., 2013b). Consistent with myofiber regeneration (Li et al., 2009b), the engraftment of transplanted MPCs was better in heterozygous (mdx;Mmp9^+/−^) mice compared to homozygous (mdx;Mmp9^−/−^) mice although both these genotypes showed better efficacy compared to mdx/Mmp9^+/+^ mice (Hindi et al., 2013b). These results suggest that increased activity of MMP-9 makes the dystrophic muscle microenvironment less permissive for the proliferation, differentiation, and fusion of MPCs. It is notable that deletion of a single allele of *Mmp9* gene reduces the protein levels of MMP-9 up to ~80% in dystrophic muscle of mdx mice (Li et al., 2009b). Higher MPC engraftment in mdx/Mmp9^+/−^ mice compared with mdx;Mmp9^−/−^ mice also suggests that a small amount of MMP-9 may be beneficial for the migration of transplanted MPCs in dystrophic muscle whereas excessive levels of MMP-9 causes pathological remodeling leading to poor engraftment (Hindi et al., 2013b). Mechanistically, it has been found that the inhibition of MMP-9 reduces the transcript levels of various proinflammatory cytokines and increases the proportion of M2 macrophages (Hindi et al., 2013b). Notch signaling, which promotes the proliferation of satellite cells (Brack et al., 2008), was increased in dystrophic muscle of mdx mice upon inhibition of MMP-9. Furthermore, MMP-9 inhibition augments the expression of the components of canonical Wnt signaling which promote myoblast fusion (Brack et al., 2008; Hindi et al., 2013b). Collectively, these studies in mdx mice have provided initial evidence that the inhibition of MMP-9 in dystrophic muscle can be a potential approach to improve myofiber regeneration and the transplantation and engraftment of MPCs.

## MMPs as biomarkers in DMD

Based on the findings that the activation of various MMPs is deregulated in muscular dystrophy, it is conceivable that some of these MMPs can serve as biomarkers for diagnosis and prognosis of muscular dystrophy. Recently, Nadarajah et al measured the levels of MMP-9, TIMP-1, and osteopontin in a small cohort of DMD patients subjected to corticosteroid therapy (Nadarajah et al., 2011). They reported that serum levels of MMP-9 and TIMP1 (but not osteopontin) were significantly higher in DMD patients compared to healthy individuals. Levels of MMP-9 (but not TIMP-1) were higher in older, non-ambulant patients, compared with ambulant patients. Moreover, a progressive age-dependent increase in the serum levels of MMP-9 was noticeable suggesting that MMP-9 is linked to the disease progression in DMD. While the study employed only 63 patients, these findings suggest that MMP-9 can be used as a potential biomarker for disease progression and monitoring therapeutic response in DMD (Nadarajah et al., 2011). Serum levels of a few MMPs (e.g., MT1-MMP, MMP-2, and MMP-9) have also been found to be increased in patients with Emery-Dreifuss muscular dystrophy and their increased levels correlate with dilated cardiomyopathy in these patients further signifies that MMPs can serve as biomarkers for diagnosis and prognosis in different types of muscular dystrophy (Niebroj-Dobosz et al., 2009).

## Concluding remarks

Studies summarized above suggest that the expression and activity of multiple MMPs are deregulated and that MMPs can be important biomarkers and therapeutic targets in muscular dystrophy. While MMPIs have been found to mitigate disease progression in mouse models, genetic studies have provided evidence that some MMPs (e.g., MMP-2 and MMP-10) may have beneficial roles in muscular dystrophy. Indeed, it is now evident that different MMPs play distinct roles in various physiological processes such as development, cell migration, and host protective mechanisms. Therefore, before considering the use of MMPIs in muscular dystrophy patients, it is critical to identify the precise role of aberrantly regulated MMPs in pathogenesis of muscular dystrophy. Intense efforts are on in different laboratories and pharmaceutical companies to develop drugs which can target a specific MMP *in vivo*.

There are also outstanding questions regarding the role of MMPs in muscular dystrophy. For example, the mechanisms of action of various MMPs in muscular dystrophy remain poorly understood. It remain unknown how the increased levels of specific MMPs cause myopathy or improve myofiber regeneration and whether they have similar roles in all types of muscular dystrophy. Understanding these mechanisms will provide additional drug targets for treatment of muscular dystrophy patients. Furthermore, the extracellular stimuli which increase the expression of specific MMPs in dystrophic muscle remain poorly defined. It is also noteworthy that most of the studies performed in animal models are short-term. It is yet to be investigated whether continued inhibition or activation of MMPs for longer duration will remain beneficial in rodent models and in higher organisms such as the golden retriever muscular dystrophy model. Nevertheless, available literature suggests that MMPs play an important role in muscular dystrophy.

## Author's contribution

Yuji Ogura and Ashok Kumar conceptualized the work. Yuji Ogura, Marjan M. Tajrishi, Shuichi Sato, Sajedah M. Hindi, and Ashok Kumar wrote the manuscript.

### Conflict of interest statement

The authors declare that the research was conducted in the absence of any commercial or financial relationships that could be construed as a potential conflict of interest.

## References

[B1] Acharya M. R. Venitz J. Figg W. D. Sparreboom A. (2004). Chemically modified tetracyclines as inhibitors of matrix metalloproteinases. Drug Resist. Updat. 7, 195–208. 10.1016/j.drup.2004.04.002 15296861

[B2] Acharyya S. Villalta S. A. Bakkar N. Bupha-Intr T. Janssen P. M. Carathers M.. (2007). Interplay of IKK/NF-kappaB signaling in macrophages and myofibers promotes muscle degeneration in Duchenne muscular dystrophy. J. Clin. Invest. 117, 889–901. 10.1172/JCI30556 17380205 PMC1821069

[B3] Ahtikoski A. M. Koskinen S. O. Virtanen P. Kovanen V. Risteli J. Takala T. E. (2003). Synthesis and degradation of type IV collagen in rat skeletal muscle during immobilization in shortened and lengthened positions. Acta Physiol. Scand. 177, 473–481. 10.1046/j.1365-201X.2003.01061.x 12648165

[B4] Allamand V. Campbell K. P. (2000). Animal models for muscular dystrophy: valuable tools for the development of therapies. Hum. Mol. Genet. 9, 2459–2467. 10.1093/hmg/9.16.2459 11005802

[B5] Asuthkar S. Velpula K. K. Nalla A. K. Gogineni V. R. Gondi C. S. Rao J. S. (2013). Irradiation-induced angiogenesis is associated with an MMP-9-miR-494-syndecan-1 regulatory loop in medulloblastoma cells. Oncogene. [Epub ahead of print]. 10.1038/onc.2013.151 23728345

[B6] Bhatnagar S. Kumar A. (2010). Therapeutic targeting of signaling pathways in muscular dystrophy. J. Mol. Med. (Berl.) 88, 155–166. 10.1007/s00109-009-0550-4 19816663 PMC2833214

[B7] Blake D. J. Weir A. Newey S. E. Davies K. E. (2002). Function and genetics of dystrophin and dystrophin-related proteins in muscle. Physiol. Rev. 82, 291–329. 10.1152/physrev.00028.2001 11917091

[B8] Bobadilla M. Sainz N. Rodriguez J. A. Abizanda G. Orbe J. de Martino A.. (2014). MMP-10 Is, Required for efficient, muscle regeneration in mouse, models of injury and muscular, dystrophy. Stem Cells 32, 447–461. 10.1002/stem.1553 24123596

[B9] Brack A. S. Conboy I. M. Conboy M. J. Shen J. Rando T. A. (2008). A temporal switch from notch to Wnt signaling in muscle stem cells is necessary for normal adult myogenesis. Cell Stem Cell 2, 50–59. 10.1016/j.stem.2007.10.006 18371421

[B10] Bramhall S. R. Hallissey M. T. Whiting J. Scholefield J. Tierney G. Stuart R. C.. (2002a). Marimastat as maintenance therapy for patients with advanced gastric cancer: a randomised trial. Br. J. Cancer 86, 1864–1870. 10.1038/sj.bjc.6600310 12085177 PMC2375430

[B11] Bramhall S. R. Rosemurgy A. Brown P. D. Bowry C. Buckels J. A. (2001). Marimastat as first-line therapy for patients with unresectable pancreatic cancer: a randomized trial. J. Clin. Oncol. 19, 3447–3455. 11481349 10.1200/JCO.2001.19.15.3447

[B12] Bramhall S. R. Schulz J. Nemunaitis J. Brown P. D. Baillet M. Buckels J. A. (2002b). A double-blind placebo-controlled, randomised study comparing gemcitabine and marimastat with gemcitabine and placebo as first line therapy in patients with advanced pancreatic cancer. Br. J. Cancer 87, 161–167. 10.1038/sj.bjc.6600446 12107836 PMC2376102

[B13] Brown P. D. (1995). Matrix metalloproteinase inhibitors: a novel class of anticancer agents. Adv. Enzyme Regul. 35, 293–301. 10.1016/0065-2571(94)00022-U 7572350

[B14] Brown P. D. (1999). Clinical studies with matrix metalloproteinase inhibitors. APMIS 107, 174–180. 10.1111/j.1699-0463.1999.tb01541.x 10190295

[B15] Campbell K. P. (1995). Three muscular dystrophies: loss of cytoskeleton-extracellular matrix linkage. Cell 80, 675–679. 10.1016/0092-8674(95)90344-5 7889563

[B16] Cerletti M. Jurga S. Witczak C. A. Hirshman M. F. Shadrach J. L. Goodyear L. J.. (2008). Highly efficient, functional engraftment of skeletal muscle stem cells in dystrophic muscles. Cell 134, 37–47. 10.1016/j.cell.2008.05.049 18614009 PMC3665268

[B17] Chakraborti S. Mandal M. Das S. Mandal A. Chakraborti T. (2003). Regulation of matrix metalloproteinases: an overview. Mol. Cell. Biochem. 253, 269–285. 10.1023/A:1026028303196 14619979

[B18] Chandler S. Miller K. M. Clements J. M. Lury J. Corkill D. Anthony D. C.. (1997). Matrix metalloproteinases, tumor necrosis factor and multiple sclerosis: an overview. J. Neuroimmunol. 72, 155–161. 10.1016/S0165-5728(96)00179-8 9042108

[B19] Chu J. W. Jones G. T. Tarr G. P. Phillips L. V. Wilkins G. T. van Rij A. M.. (2011). Plasma active matrix metalloproteinase 9 associated to diastolic dysfunction in patients with coronary artery disease. Int. J. Cardiol. 147, 336–338. 10.1016/j.ijcard.2010.12.093 21276625

[B20] Cohn R. D. Campbell K. P. (2000). Molecular basis of muscular dystrophies. Muscle Nerve 23, 1456–1471. 10.1002/1097-4598(200010)23:10%3C1456::AID-MUS2%3E3.3.CO;2-K 11003781

[B21] Corry D. B. Kiss A. Song L. Z. Song L. Xu J. Lee S. H.. (2004). Overlapping and independent contributions of MMP2 and MMP9 to lung allergic inflammatory cell egression through decreased CC chemokines. FASEB J. 18, 995–997. 10.1096/fj.03-1412fje 15059974 PMC2771179

[B22] Dahiya S. Bhatnagar S. Hindi S. M. Jiang C. Paul P. K. Kuang S.. (2011b). Elevated levels of active matrix metalloproteinase-9 cause hypertrophy in skeletal muscle of normal and dystrophin-deficient mdx mice. Hum. Mol. Genet. 20, 4345–4359. 10.1093/hmg/ddr362 21846793 PMC3196885

[B23] Dahiya S. Givvimani S. Bhatnagar S. Qipshidze N. Tyagi S. C. Kumar A. (2011a). Osteopontin-stimulated expression of matrix metalloproteinase-9 causes cardiomyopathy in the mdx model of Duchenne muscular dystrophy. J. Immunol. 187, 2723–2731. 10.4049/jimmunol.1101342 21810612 PMC3159792

[B24] Darabi R. Santos F. N. Perlingeiro R. C. (2008). The therapeutic potential of embryonic and adult stem cells for skeletal muscle regeneration. Stem Cell Rev. 4, 217–225. 10.1007/s12015-008-9023-3 18607783

[B25] Das S. Mandal M. Chakraborti T. Mandal A. Chakraborti S. (2003). Structure and evolutionary aspects of matrix metalloproteinases: a brief overview. Mol. Cell. Biochem. 253, 31–40. 10.1023/A:1026093016148 14619953

[B26] Davies J. E. Wang L. Garcia-Oroz L. Cook L. J. Vacher C. O'Donovan D. G.. (2005). Doxycycline attenuates and delays toxicity of the oculopharyngeal muscular dystrophy mutation in transgenic mice. Nat. Med. 11, 672–677. 10.1038/nm1242 15864313

[B27] Delfin D. A. Zang K. E. Schill K. E. Patel N. T. Janssen P. M. Raman S. V.. (2012). Cardiomyopathy in the dystrophin/utrophin-deficient mouse model of severe muscular dystrophy is characterized by dysregulation of matrix metalloproteinases. Neuromuscul. Disord. 22, 1006–1114. 10.1016/j.nmd.2012.05.002 22749475 PMC3484217

[B28] Dezawa M. Ishikawa H. Itokazu Y. Yoshihara T. Hoshino M. Takeda S.. (2005). Bone marrow stromal cells generate muscle cells and repair muscle degeneration. Science 309, 314–317. 10.1126/science.1110364 16002622

[B29] Dove A. (2002). MMP inhibitors: glimmers of hope amidst clinical failures. Nat. Med. 8:95. 10.1038/nm0202-95 11821877

[B30] Ducharme A. Frantz S. Aikawa M. Rabkin E. Lindsey M. Rohde L. E.. (2000). Targeted deletion of matrix metalloproteinase-9 attenuates left ventricular enlargement and collagen accumulation after experimental myocardial infarction. J. Clin. Invest. 106, 55–62. 10.1172/JCI8768 10880048 PMC517910

[B31] Emery A. E. (2002). The muscular dystrophies. Lancet 359, 687–695. 10.1016/S0140-6736(02)07815-7 11879882

[B32] Fernandes K. S. Brum D. G. Sandrim V. C. Guerreiro C. T. Barreira A. A. Tanus-Santos J. E. (2009). Matrix metalloproteinase-9 genotypes and haplotypes are associated with multiple sclerosis and with the degree of disability of the disease. J. Neuroimmunol. 214, 128–131. 10.1016/j.jneuroim.2009.07.004 19631393

[B33] Ferretti G. Fabi A. Carlini P. Papaldo P. Cordiali Fei P. Di Cosimo S.. (2005). Zoledronic-acid-induced circulating level modifications of angiogenic factors, metalloproteinases and proinflammatory cytokines in metastatic breast cancer patients. Oncology 69, 35–43. 10.1159/000087286 16088233

[B34] Fingleton B. (2007). Matrix metalloproteinases as valid clinical targets. Curr. Pharm. Des. 13, 333–346. 10.2174/138161207779313551 17313364

[B35] Fingleton B. (2008). MMPs as therapeutic targets–still a viable option? Semin. Cell Dev. Biol. 19, 61–68. 10.1016/j.semcdb.2007.06.006 17693104 PMC2677300

[B36] Fukuda Y. Ishizaki M. Kudoh S. Kitaichi M. Yamanaka N. (1998). Localization of matrix metalloproteinases-1, -2, and -9 and tissue inhibitor of metalloproteinase-2 in interstitial lung diseases. Lab. Invest. 78, 687–698. 9645759

[B37] Fukushima K. Nakamura A. Ueda H. Yuasa K. Yoshida K. Takeda S.. (2007). Activation and localization of matrix metalloproteinase-2 and -9 in the skeletal muscle of the muscular dystrophy dog (CXMDJ). BMC Musculoskelet. Disord. 8:54. 10.1186/1471-2474-8-54 17598883 PMC1929071

[B38] Gargioli C. Coletta M. De Grandis F. Cannata S. M. Cossu G. (2008). PlGF-MMP-9-expressing cells restore microcirculation and efficacy of cell therapy in aged dystrophic muscle. Nat. Med. 14, 973–978. 10.1038/nm.1852 18660817

[B39] Giraudo E. Inoue M. Hanahan D. (2004). An amino-bisphosphonate targets MMP-9-expressing macrophages and angiogenesis to impair cervical carcinogenesis. J. Clin. Invest. 114, 623–633. 10.1172/JCI200422087 15343380 PMC514591

[B40] Girgenrath M. Beermann M. L. Vishnudas V. K. Homma S. Miller J. B. (2009). Pathology is alleviated by doxycycline in a laminin-alpha2-null model of congenital muscular dystrophy. Ann. Neurol. 65, 47–56. 10.1002/ana.21523 19086074 PMC2639627

[B41] Grams F. Crimmin M. Hinnes L. Huxley P. Pieper M. Tschesche H.. (1995). Structure determination and analysis of human neutrophil collagenase complexed with a hydroxamate inhibitor. Biochemistry 34, 14012–14020. 10.1021/bi00043a007 7577999

[B42] Gum R. Lengyel E. Juarez J. Chen J. H. Sato H. Seiki M.. (1996). Stimulation of 92-kDa gelatinase B promoter activity by ras is mitogen-activated protein kinase kinase 1-independent and requires multiple transcription factor binding sites including closely spaced PEA3/ets and AP-1 sequences. J. Biol. Chem. 271, 10672–10680. 10.1074/jbc.271.18.10672 8631874

[B43] Halade G. V. Jin Y. F. Lindsey M. L. (2013). Matrix metalloproteinase (MMP)-9: a proximal biomarker for cardiac remodeling and a distal biomarker for inflammation. Pharmacol. Ther. 139, 32–40. 10.1016/j.pharmthera.2013.03.009 23562601 PMC3660444

[B44] Haslett J. N. Sanoudou D. Kho A. T. Bennett R. R. Greenberg S. A. Kohane I. S.. (2002). Gene expression comparison of biopsies from Duchenne muscular dystrophy (DMD) and normal skeletal muscle. Proc. Natl. Acad. Sci. U.S.A. 99, 15000–15005. 10.1073/pnas.192571199 12415109 PMC137534

[B45] Hindi S. M. Shin J. Ogura Y. Li H. Kumar A. (2013b). Matrix metalloproteinase-9 inhibition improves proliferation and engraftment of myogenic cells in dystrophic muscle of mdx mice. PLoS ONE 8:e72121. 10.1371/journal.pone.0072121 23977226 PMC3744489

[B46] Hindi S. M. Tajrishi M. M. Kumar A. (2013a). Signaling mechanisms in mammalian myoblast fusion. Sci. Signal. 6:re2. 10.1126/scisignal.2003832 23612709 PMC3724417

[B47] Hnia K. Gayraud J. Hugon G. Ramonatxo M. De La Porte S. Matecki S.. (2008). L-arginine decreases inflammation and modulates the nuclear factor-kappaB/matrix metalloproteinase cascade in mdx muscle fibers. Am. J. Pathol. 172, 1509–1519. 10.2353/ajpath.2008.071009 18458097 PMC2408412

[B48] Hnia K. Hugon G. Rivier F. Masmoudi A. Mercier J. Mornet D. (2007). Modulation of p38 mitogen-activated protein kinase cascade and metalloproteinase activity in diaphragm muscle in response to free radical scavenger administration in dystrophin-deficient Mdx mice. Am. J. Pathol. 170, 633–643. 10.2353/ajpath.2007.060344 17255331 PMC1851881

[B49] Hoffman E. P. Brown R. H. Jr. Kunkel L. M. (1987). Dystrophin: the protein product of the Duchenne muscular dystrophy locus. Cell 51, 919–928. 10.1016/0092-8674(87)90579-4 3319190

[B50] Kherif S. Lafuma C. Dehaupas M. Lachkar S. Fournier J. G. Verdiere-Sahuque M.. (1999). Expression of matrix metalloproteinases 2 and 9 in regenerating skeletal muscle: a study in experimentally injured and mdx muscles. Dev. Biol. 205, 158–170. 10.1006/dbio.1998.9107 9882504

[B51] Kjaer M. (2004). Role of extracellular matrix in adaptation of tendon and skeletal muscle to mechanical loading. Physiol. Rev. 84, 649–698. 10.1152/physrev.00031.2003 15044685

[B52] Koskinen S. O. Kjaer M. Mohr T. Sorensen F. B. Suuronen T. Takala T. E. (2000). Type IV collagen and its degradation in paralyzed human muscle: effect of functional electrical stimulation. Muscle Nerve 23, 580–589. 10.1002/(SICI)1097-4598(200004)23:4%3C580::AID-MUS18%3E3.0.CO;2-4 10716770

[B53] Kumar A. Bhatnagar S. Kumar A. (2010). Matrix metalloproteinase inhibitor batimastat alleviates pathology and improves skeletal muscle function in dystrophin-deficient mdx mice. Am. J. Pathol. 177, 248–260. 10.2353/ajpath.2010.091176 20472898 PMC2893668

[B54] Kumar A. Boriek A. M. (2003). Mechanical stress activates the nuclear factor-kappaB pathway in skeletal muscle fibers: a possible role in Duchenne muscular dystrophy. FASEB J. 17, 386–396. 10.1096/fj.02-0542com 12631578

[B55] Kumar A. Khandelwal N. Malya R. Reid M. B. Boriek A. M. (2004). Loss of dystrophin causes aberrant mechanotransduction in skeletal muscle fibers. FASEB J. 18, 102–113. 10.1096/fj.03-0453com 14718391

[B56] Ladwig G. P. Robson M. C. Liu R. Kuhn M. A. Muir D. F. Schultz G. S. (2002). Ratios of activated matrix metalloproteinase-9 to tissue inhibitor of matrix metalloproteinase-1 in wound fluids are inversely correlated with healing of pressure ulcers. Wound Repair Regen. 10, 26–37. 10.1046/j.1524-475X.2002.10903.x 11983004

[B57] Lewis M. P. Machell J. R. Hunt N. P. Sinanan A. C. Tippett H. L. (2001). The extracellular matrix of muscle–implications for manipulation of the craniofacial musculature. Eur. J. Oral Sci. 109, 209–221. 10.1034/j.1600-0722.2001.00021.x 11531066

[B58] Li H. Malhotra S. Kumar A. (2008). Nuclear factor-kappa B signaling in skeletal muscle atrophy. J. Mol. Med. (Berl.) 86, 1113–1126. 10.1007/s00109-008-0373-8 18574572 PMC2597184

[B59] Li H. Mittal A. Makonchuk D. Y. Bhatnagar S. Kumar A. (2009b). Matrix metalloproteinase-9 Inhibition ameliorates pathogenesis and improves skeletal muscle regeneration in muscular dystrophy. Hum. Mol. Genet. 18, 2584–2598. 10.1093/hmg/ddp191 19401296 PMC2701330

[B60] Li H. Mittal A. Paul P. K. Kumar M. Srivastava D. S. Tyagi S. C.. (2009a). Tumor necrosis factor-related weak inducer of apoptosis augments matrix metalloproteinase 9 (MMP-9) production in skeletal muscle through the activation of nuclear factor-kappaB-inducing kinase and p38 mitogen-activated protein kinase: a potential role of MMP-9 in myopathy. J. Biol. Chem. 284, 4439–4450. 10.1074/jbc.M805546200 19074147 PMC2640955

[B61] Macaulay V. M. O'Byrne K. J. Saunders M. P. Braybrooke J. P. Long L. Gleeson F.. (1999). Phase, I study of intrapleural batimastat (BB-94), a matrix metalloproteinase inhibitor, in the treatment of malignant pleural effusions. Clin. Cancer Res. 5, 513–520. 10100701

[B62] Martin M. D. Carter K. J. Jean-Philippe S. R. Chang M. Mobashery S. Thiolloy S.. (2008). Effect of ablation or inhibition of stromal matrix metalloproteinase-9 on lung metastasis in a breast cancer model is dependent on genetic background. Cancer Res. 68, 6251–6259. 10.1158/0008-5472.CAN-08-0537 18676849 PMC2789265

[B63] Matsumura K. Zhong D. Saito F. Arai K. Adachi K. Kawai H.. (2005). Proteolysis of beta-dystroglycan in muscular diseases. Neuromuscul. Disord. 15, 336–341. 10.1016/j.nmd.2005.01.007 15833425

[B64] Michaluk P. Kolodziej L. Mioduszewska B. Wilczynski G. M. Dzwonek J. Jaworski J.. (2007). Beta-dystroglycan as a target for MMP-9, in response to enhanced neuronal activity. J. Biol. Chem. 282, 16036–16041. 10.1074/jbc.M700641200 17426029

[B65] Miyazaki D. Nakamura A. Fukushima K. Yoshida K. Takeda S. Ikeda S. I. (2011). Matrix metalloproteinase-2 ablation in dystrophin-deficient mdx muscle reduces angiogenesis resulting in impaired growth of regenerated muscle fibers. Hum. Mol. Genet. 20, 1787–1799. 10.1093/hmg/ddr062 21320869

[B66] Mott J. D. Werb Z. (2004). Regulation of matrix biology by matrix metalloproteinases. Curr. Opin. Cell Biol. 16, 558–564. 10.1016/j.ceb.2004.07.010 15363807 PMC2775446

[B67] Munoz-Valle J. F. Vazquez-Del Mercado M. Garcia-Iglesias T. Orozco-Barocio G. Bernard-Medina G. Martinez-Bonilla G.. (2003). T(H)1/T(H)2 cytokine profile, metalloprotease-9 activity and hormonal status in pregnant rheumatoid arthritis and systemic lupus erythematosus patients. Clin. Exp. Immunol. 131, 377–384. 10.1046/j.1365-2249.2003.02059.x 12562402 PMC1808625

[B68] Nadarajah V. D. van Putten M. Chaouch A. Garrood P. Straub V. Lochmuller H.. (2011). Serum matrix metalloproteinase-9 (MMP-9) as a biomarker for monitoring disease progression in Duchenne muscular dystrophy (DMD). Neuromuscul. Disord. 21, 569–578. 10.1016/j.nmd.2011.05.011 21724396

[B69] Nagase H. Woessner J. F. Jr. (1999). Matrix metalloproteinases. J. Biol. Chem. 274, 21491–21494. 10.1074/jbc.274.31.21491 10419448

[B70] Nair R. R. Boyd D. D. (2005). Expression cloning of novel regulators of 92 kDa type IV collagenase expression. Biochem. Soc. Trans. 33(Pt 5), 1135–1136. 10.1042/BST20051135 16246065

[B71] Nemunaitis J. Poole C. Primrose J. Rosemurgy A. Malfetano J. Brown P.. (1998). Combined analysis of studies of the effects of the matrix metalloproteinase inhibitor marimastat on serum tumor markers in advanced cancer: selection of a biologically active and tolerable dose for longer-term studies. Clin Cancer Res. 4, 1101–1109. 9607566

[B72] Niebroj-Dobosz I. Madej-Pilarczyk A. Marchel M. Sokolowska B. Hausmanowa-Petrusewicz I. (2009). Matrix metalloproteinases in serum of Emery-Dreifuss muscular dystrophy patients. Acta Biochim. Pol. 56, 717–722. 19997654

[B73] Page-McCaw A. Ewald A. J. Werb Z. (2007). Matrix metalloproteinases and the regulation of tissue remodelling. Nat. Rev. Mol. Cell. Biol. 8, 221–233. 10.1038/nrm2125 17318226 PMC2760082

[B74] Parsons S. L. Watson S. A. Steele R. J. (1997). Phase, I/II trial of batimastat, a matrix metalloproteinase inhibitor, in patients with malignant ascites. Eur. J. Surg. Oncol. 23, 526–531. 10.1016/S0748-7983(97)93077-8 9484924

[B75] Percival J. M. Whitehead N. P. Adams M. E. Adamo C. M. Beavo J. A. Froehner S. C. (2012). Sildenafil reduces respiratory muscle weakness and fibrosis in the mdx mouse model of Duchenne muscular dystrophy. J. Pathol. 228, 77–87. 10.1002/path.4054 22653783 PMC4067455

[B76] Pereira J. A. Matsumura C. Y. Minatel E. Marques M. J. Santo Neto H. (2014). Understanding the beneficial effects of doxycycline on the dystrophic phenotype of the mdx mouse. Muscle Nerve. [Epub ahead of print]. 10.1002/mus.24177 24435758

[B77] Pereira J. A. Taniguti A. P. Matsumura C. Marques M. J. Neto H. S. (2012). Doxycycline ameliorates the dystrophic phenotype of skeletal and cardiac muscles in mdx mice. Muscle Nerve 46, 400–406. 10.1002/mus.23331 22907231

[B78] Pichavant C. Gargioli C. Tremblay J. P. (2011). Intramuscular, transplantation of Muscle, precursor cells over-expressing MMP-9 improves transplantation, success. PLoS Curr. 3:RRN1275. 10.1371/currents.RRN1275 22052037 PMC3206262

[B79] Porter J. D. Khanna S. Kaminski H. J. Rao J. S. Merriam A. P. Richmonds C. R.. (2002). A chronic inflammatory response dominates the skeletal muscle molecular signature in dystrophin-deficient mdx mice. Hum. Mol. Genet. 11, 263–272. 10.1093/hmg/11.3.263 11823445

[B80] Price F. D. Kuroda K. Rudnicki M. A. (2007). Stem cell based therapies to treat muscular dystrophy. Biochim. Biophys. Acta 1772, 272–283. 10.1016/j.bbadis.2006.08.011 17034994

[B81] Prins K. W. Humston J. L. Mehta A. Tate V. Ralston E. Ervasti J. M. (2009). Dystrophin is a microtubule-associated protein. J. Cell Biol. 186, 363–369. 10.1083/jcb.200905048 19651889 PMC2728405

[B82] Qu Z. Balkir L. van Deutekom J. C. Robbins P. D. Pruchnic R. Huard J. (1998). Development of approaches to improve cell survival in myoblast transfer therapy. J. Cell Biol. 142, 1257–1267. 10.1083/jcb.142.5.1257 9732286 PMC2149359

[B83] Ram M. Sherer Y. Shoenfeld Y. (2006). Matrix metalloproteinase-9 and autoimmune diseases. J. Clin. Immunol. 26, 299–307. 10.1007/s10875-006-9022-6 16652230

[B84] Rando T. A. (2001). The dystrophin-glycoprotein complex, cellular signaling, and the regulation of cell survival in the muscular dystrophies. Muscle Nerve 24, 1575–1594. 10.1002/mus.1192 11745966

[B85] Rasmussen H. S. McCann P. P. (1997). Matrix metalloproteinase inhibition as a novel anticancer strategy: a review with special focus on batimastat and marimastat. Pharmacol. Ther. 75, 69–75. 10.1016/S0163-7258(97)00023-5 9364582

[B86] Rayment E. A. Upton Z. Shooter G. K. (2008). Increased matrix metalloproteinase-9 (MMP-9) activity observed in chronic wound fluid is related to the clinical severity of the ulcer. Br. J. Dermatol. 158, 951–961. 10.1111/j.1365-2133.2008.08462.x 18284390

[B87] Renkiewicz R. Qiu L. Lesch C. Sun X. Devalaraja R. Cody T.. (2003). Broad-spectrum matrix metalloproteinase inhibitor marimastat-induced musculoskeletal side effects in rats. Arthritis Rheum. 48, 1742–1749. 10.1002/art.11030 12794843

[B88] Roma J. Munell F. Fargas A. Roig M. (2004). Evolution of pathological changes in the gastrocnemius of the mdx mice correlate with utrophin and beta-dystroglycan expression. Acta Neuropathol. 108, 443–452. 10.1007/s00401-004-0908-1 15365724

[B89] Sampaolesi M. Blot S. D'Antona G. Granger N. Tonlorenzi R. Innocenzi A.. (2006). Mesoangioblast stem cells ameliorate muscle function in dystrophic dogs. Nature 444, 574–579. 10.1038/nature05282 17108972

[B90] Sanes J. R. (2003). The basement membrane/basal lamina of skeletal muscle. J. Biol. Chem. 278, 12601–12604. 10.1074/jbc.R200027200 12556454

[B91] Shi H. Verma M. Zhang L. Dong C. Flavell R. A. Bennett A. M. (2013). Improved regenerative myogenesis and muscular dystrophy in mice lacking Mkp5. J. Clin. Invest. 123, 2064–2077. 10.1172/JCI64375 23543058 PMC3635719

[B92] Shin J. Tajrishi M. M. Ogura Y. Kumar A. (2013). Wasting mechanisms in muscular dystrophy. Int. j. Biochem. cell Biol. 45, 2266–2279. 10.1016/j.biocel.2013.05.001 23669245 PMC3759654

[B93] Sohar I. Laszlo A. Gaal K. Mechler F. (1988). Cysteine and metalloproteinase activities in serum of Duchenne muscular dystrophic genotypes. Biol. Chem. Hoppe-Seyler 369(Suppl.), 277–229. 3202967

[B94] Srivastava A. K. Qin X. Wedhas N. Arnush M. Linkhart T. A. Chadwick R. B.. (2007). Tumor necrosis factor-alpha augments matrix metalloproteinase-9 production in skeletal muscle cells through the activation of transforming growth factor-beta-activated kinase 1 (TAK1)-dependent signaling pathway. J. Biol. Chem. 282, 35113–35124. 10.1074/jbc.M705329200 17897957 PMC4154379

[B95] Tajrishi M. M. Zheng T. S. Burkly L. C. Kumar A. (2014). The TWEAK-Fn14 pathway: a potent regulator of skeletal muscle biology in health and disease. Cytokine Growth Factor Rev. [Epub ahead of print]. 10.1016/j.cytogfr.2013.12.004 24444596 PMC3999262

[B96] Taniguti A. P. Matsumura C. Y. Rodrigues-Simioni L. Santo Neto H. Marques M. J. (2012). Suramin affects metalloproteinase-9 activity and increases beta-dystroglycan levels in the diaphragm of the dystrophin-deficient mdx mouse. Muscle Nerve 46, 810–813. 10.1002/mus.23468 23055317

[B97] Turpeenniemi-Hujanen T. Thorgeirsson U. P. Hart I. R. Grant S. S. Liotta L. A. (1985). Expression of collagenase IV (basement membrane collagenase) activity in murine tumor cell hybrids that differ in metastatic potential. J. Natl. Cancer Inst. 75, 99–103. 2989606

[B98] Tyagi S. C. Kumar S. G. Haas S. J. Reddy H. K. Voelker D. J. Hayden M. R.. (1996). Post-transcriptional regulation of extracellular matrix metalloproteinase in human heart end-stage failure secondary to ischemic cardiomyopathy. J. Mol. Cell. Cardiol. 28, 1415–1428. 10.1006/jmcc.1996.0132 8841929

[B99] Vetrone S. A. Montecino-Rodriguez E. Kudryashova E. Kramerova I. Hoffman E. P. Liu S. D.. (2009). Osteopontin promotes fibrosis in dystrophic mouse muscle by modulating immune cell subsets and intramuscular TGF-beta. J. Clin. Invest. 119, 1583–1594. 10.1172/JCI37662 19451692 PMC2689112

[B100] von Moers A. Zwirner A. Reinhold A. Bruckmann O. van Landeghem F. Stoltenburg-Didinger G.. (2005). Increased mRNA expression of tissue inhibitors of metalloproteinase-1 and -2 in Duchenne muscular dystrophy. Acta Neuropathol. 109, 285–293. 10.1007/s00401-004-0941-0 15616792

[B101] Vu T. H. Werb Z. (2000). Matrix metalloproteinases: effectors of development and normal physiology. Genes Dev. 14, 2123–2133. 10.1101/gad.815400 10970876

[B102] Wilson W. R. Evans J. Bell P. R. Thompson M. M. (2005). HMG-CoA reductase inhibitors (statins) decrease MMP-3 and MMP-9 concentrations in abdominal aortic aneurysms. Eur. J. Vasc. Endovasc. Surg. 30, 259–262. 10.1016/j.ejvs.2005.02.044 16009575

[B103] Wu D. Huang P. Wang L. Zhou Y. Pan H. Qu P. (2013). MicroRNA-143 inhibits cell migration and invasion by targeting matrix metalloproteinase 13 in prostate cancer. Mol. Med. Rep. 8, 626–630. 10.3892/mmr.2013.1501 23732700

[B104] Xu D. McKee C. M. Cao Y. Ding Y. Kessler B. M. Muschel R. J. (2010). Matrix metalloproteinase-9 regulates tumor cell invasion through cleavage of protease nexin-1. Cancer Res. 70, 6988–6998. 10.1158/0008-5472.CAN-10-0242 20736374 PMC3272441

[B105] Xu N. Zhang L. Meisgen F. Harada M. Heilborn J. Homey B.. (2012). MicroRNA-125b down-regulates matrix metallopeptidase 13 and inhibits cutaneous squamous cell carcinoma cell proliferation, migration, and invasion. J. Biol. Chem. 287, 29899–29908. 10.1074/jbc.M112.391243 22782903 PMC3436131

[B106] Yasuda S. Miyazaki S. Kinoshita H. Nagaya N. Kanda M. Goto Y.. (2007). Enhanced cardiac production of matrix metalloproteinase-2 and -9 and its attenuation associated with pravastatin treatment in patients with acute myocardial infarction. Clin. Sci. 112, 43–49. 10.1042/CS20060110 16939410

[B107] Yu Q. Stamenkovic I. (2000). Cell surface-localized matrix metalloproteinase-9 proteolytically activates TGF-beta and promotes tumor invasion and angiogenesis. Genes Dev. 14, 163–176. 10.1101/gad.14.2.163 10652271 PMC316345

[B108] Zanotti S. Gibertini S. Mora M. (2010). Altered production of extra-cellular matrix components by muscle-derived Duchenne muscular dystrophy fibroblasts before and after TGF-beta1 treatment. Cell Tissue Res. 339, 397–410. 10.1007/s00441-009-0889-4 19902258

[B109] Zanotti S. Saredi S. Ruggieri A. Fabbri M. Blasevich F. Romaggi S.. (2007). Altered extracellular matrix transcript expression and protein modulation in primary Duchenne muscular dystrophy myotubes. Matrix Biol. 26, 615–624. 10.1016/j.matbio.2007.06.004 17662584

[B110] Zhong D. Saito F. Saito Y. Nakamura A. Shimizu T. Matsumura K. (2006). Characterization of the protease activity that cleaves the extracellular domain of beta-dystroglycan. Biochem. Biophys. Res. Commun. 345, 867–871. 10.1016/j.bbrc.2006.05.004 16701552

